# RALF proteins—a monitoring hub for regulating salinity tolerance in plants

**DOI:** 10.3389/fpls.2024.1365133

**Published:** 2025-01-03

**Authors:** Liping Huang, Xing Liu, Qianqian Wang, Wen Chen, Wenxuan Fu, Yongjun Guo

**Affiliations:** ^1^ International Research Center for Environmental Membrane Biology, College of Food Science and Engineering, Foshan University, Foshan, China; ^2^ Foshan ZhiBao Ecological Technology Co. Ltd, Foshan, Guangdong, China

**Keywords:** salinity tolerance, Ca 2+ signaling, PM-depolarization, root activity, H + -ATPase, hormonal regulation, ROS production

The rapid alkalinization factor (RALF) polypeptide is a small (composed of 49 amino acids) molecule that was originally found in tobacco (*Nicotiana tabacum*) leaves (Pearce et al., 2001) and has been studied for over 20 years as a plant development regulator (Blackburn et al., 2020). RALF peptides are widely distributed in the plant kingdom and regulate several key developmental processes including root development (Wu et al., 2007; Murphy et al., 2016), pollen growth (Ge et al., 2017), and salinity stress (SS) tolerance (Zhao et al., 2018). However, the evolutionary analysis of homologous genes encoding RALF precursor proteins showed that RALF initially had the function of accelerating apical growth, and ultimately differentiated into new functions in vascular plants through multiple tandem replication events (Cao and Shi, 2012; Sharma et al., 2016; Campbell and Turner, 2017; Ginanjar et al., 2022). The functional differences in RALFs drive complex morphogenesis in land plants and facilitate other novel processes (Liu et al., 2024).

Structural analysis of the RALF proteins shows that RALFs consist of a single peptide and a mature RALF peptide (**Figure 1A**). The mature peptide has relatively conserved domains and is mainly divided into four major clades and two distinct functional subgroups. The mature RALF sequence’s N- and C-terminal regions can interact with different receptor proteins, thus forming different complexes by producing different conformational changes. For instance, the N-terminus of *AtRALF23* formed the α-helix in the complex, which binds to the large surface groove on LLG2, while the C-terminus can bind to the extracellular domain of FERONIA (FER) (Xiao et al., 2019). In the RALF-LLG- BUDDHA’S PAPER SEAL 1/2/ANXUR 1/2 complex, the N-terminal region of RALF4 is necessary for its biological function (Ge et al., 2019), indicating that RALFs can bind to different proteins to form different complexes.

**Figure 1:**
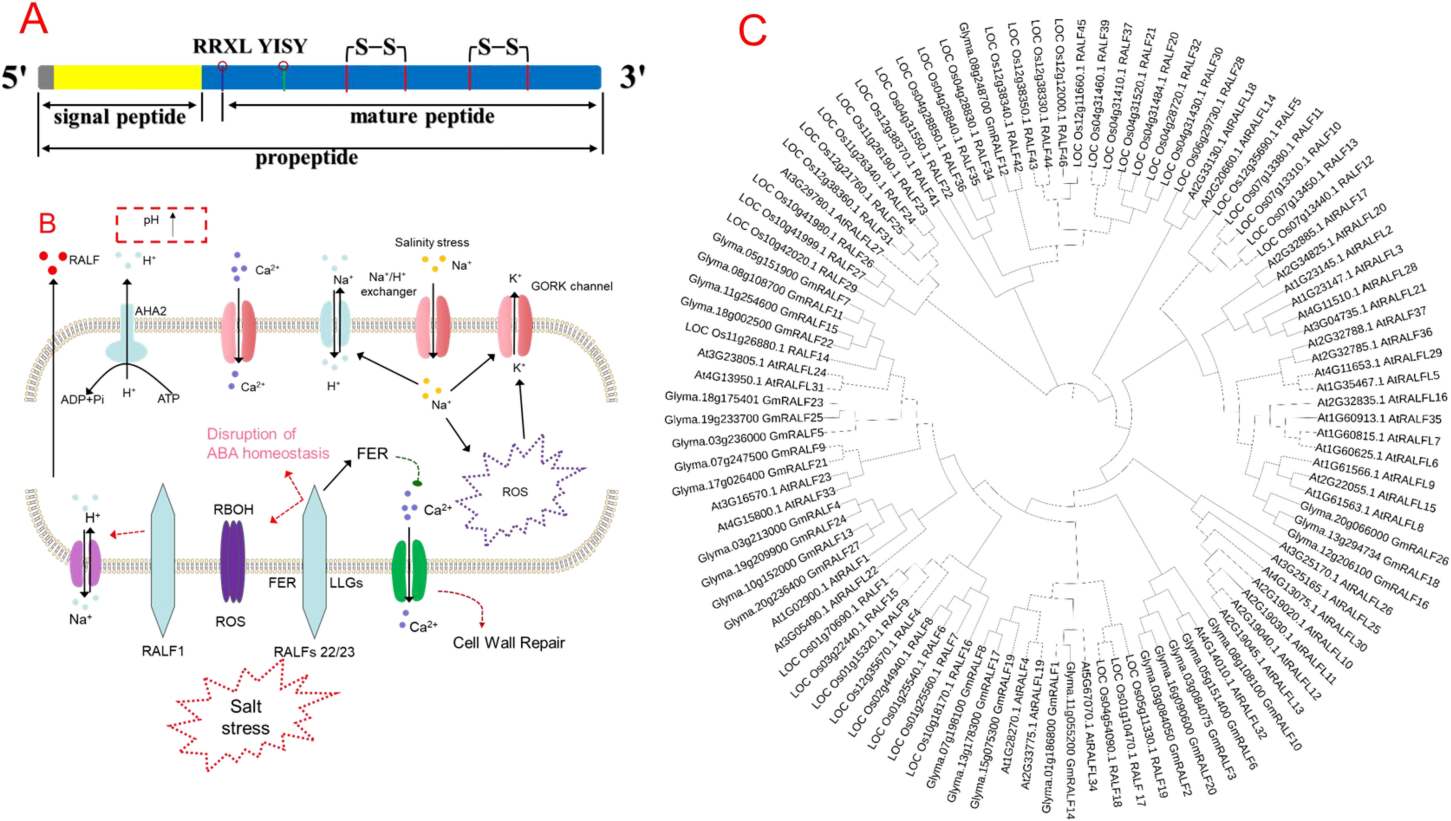
**(A)** Structural composition of rapid alkalinization factor (RALF) peptide, **(B)** role of RALF in regulating salinity tolerance in plants, and **(C)** phylogenetic tree of different RALF genes in plant species. The lower side of the cell shows the activity of RALFs under salt stress; NaCl-induced Ca^2+^ is sensed by the extracellular domain of FERONIA (FER) and co-receptor LLGs, which also senses the cell wall perturbation and initiates cell wall repair (Feng et al., 2018). Higher Na^+^ entry into cytosol induces cell wall perturbation, dissociates RALF22/23 from LRXs, and promotes RALF22/23-induced internalization of FER, and finally, RALF inhibits the signal transduction ability of FER (Zhao et al., 2018). Moreover, RALF complex with LRX and FER induces cell death under salt stress through loss of ABA homeostasis, higher Na^+^ accumulation, and ROS production mediated by the Respiratory burst oxidase homolog (RBOH) gene (Zhao et al., 2021). Phylogenetic tree of different RALF genes in Arabidopsis, rice, and soybean. We considered one model plant species (*Arabidopsis thaliana* L.), one cereal (rice—*Oryza sativa* L.), and one legume (soybean—*Glycine max* L.). We first found the number of RALF genes; for example, 27 soybeans, 41 rice, and 34 Arabidopsis RALF protein sequences were downloaded from the phytozome (https://phytozome-next.jgi.doe.gov/), rice Genomics Network (https://rice.uga.edu/cgi-bin/gbrowse/rice/), and TAIR (www.arabidopsis.org/) databases, respectively. The phylogenetic tree was produced using MEGA 11 software via the Neighbor-Joining (NJ) method with 1,000 bootstrap replicates.

Many RALF peptides can cause extracellular alkalization by increasing the extracellular pH. For instance, in H^+^-ATPase 2 (AHA2) protein, the phosphorylation of Thr881 and Thr947 activated H^+^ transport (Li et al., 2022b), while the RALF–FER interaction phosphorylates plasma membrane (PM) H^+^-ATPase at Ser899 and then mediates the inhibition of proton transport (Haruta et al., 2014), indicating that the inhibition of H^+^ pump activity is important for the increasing extracellular pH caused by RALF.

SS is a worldwide dilemma and improving salinity tolerance (ST) in plants is highly complex due to the involvement of several key players in regulating ST in plants (Khan et al., 2020; Tanveer and Ahmed, 2020). The SS reduces plant growth by inducing membrane depolarization, which later increases ROS production in the cytosol and activated voltage-gated and ROS-activated K^+^-outward rectifying channel and transporter at the PM (**Figure 1B**) (Wegner et al., 2011; Shabala et al., 2015). Thus, in this context, the activation of PM-H^+^-ATPase and Ca^2+^ signaling is critical in regulating cytosolic K^+^ homeostasis and ST in plants.

Compared with alkalization, higher activation of H^+^-ATPase is required to reduce the SS-induced activation of voltage-gated K^+^ outward rectifying channels (e.g., GORK) at the PM (Shabala S. et al., 2016). SS results in a significant membrane depolarization leading to a considerable disturbance in cell ionic balance and metabolism. Plants’ ability to maintain highly negative membrane potential (MP) values has been firmly associated with their tolerance to SS (Chen et al., 2007; Bose et al., 2015; Chakraborty et al., 2016). In this context, RALF proteins are important for alkalization and may play an important role in regulating PM-H^+^-ATPase to regulate ST in plants. RALF regulates the activity of H^+^-ATPase (Gjetting et al., 2020), which later imposes a massive implication to the regulation of plant ionic homeostasis via controlling cell MP at the PM. Though H^+^-ATPase activity is critical in determining cell MP (Palmgren and Nissen, 2011), more negative MPs are required for the operation of voltage-gated ion channels and ion transporters. The H^+^ gradients between extracellular and intracellular space create an H^+^ motive force for the secondary active transport of other ions (e.g., K^+^, NH_4_^+^, NO_3_^-^; PO_4_^2-^; SO_4_^2-^) via H^+^-coupled co-transport systems (Shabala S. et al., 2016). Thus, RALF-induced modulation of H^+^-ATPase activity may be an essential factor in controlling cellular MP and, ultimately, cell metabolism under stress conditions. However, it has been argued that higher H^+^-ATPase activity may lead to ATP reduction in the cells, which later affects the ability of plants to survive under SS (Rubio et al., 2020). Thus, tight regulation between the activation of RALF-mediated H^+^-ATPase and ATP reduction is required. However, there is also less evidence to prove that RALF as an upstream inhibitor of H^+^-ATPase blocks some channels or transporters (e.g., SOS1 and HAK family) that are driven by H^+^ gradient. Moreover, owing to the diverse ability of RALFs to interact with other receptor proteins, it can be suggested that different RALFs may affect different signal pathways to activate H^+^ transporters/channels in the cell and regulate ST in plants. The LRR domains of LRX3 and LRX4 can interact with both *AtRALF22*/23 and FER to regulate salt stress response (Zhao et al., 2018). Recently, Gjetting et al. (2020) showed that the H^+^ pump activity was increased three times after the application of 1 µM RALF33/36, while the *aha* mutant was hypersensitive to *AtRALF1*, which was some unknown H^+^ transporters or channels that led to the net influx of H^+^ in the cytoplasm and an increase in extracellular pH in highly possible (Li et al., 2022a). However, how cells accurately perceive the extracellular environmental pH and release RALF to regulate it is a problem that needs to be solved.

SS also triggers apoplastic alkalization and thereby inhibits plant growth (Kesten et al., 2019); however, it can also induce the formation of mature RALFs (Zhao et al., 2018). In two halophytes, higher activation of H^+^-ATPases under SS contributes to Na^+^ efflux from cytosol and low apoplastic pH associated with higher Na^+^/H^+^ exchanger at PM (Bose et al., 2015). Such SS-induced apoplastic alkalization could later be mediated by RALF–FER–AHA2 and apoplastic acidification is important for ST. This aspect needs to be examined in future studies.

Ca^2+^ being an important component of the cell wall (CW) and membrane structure (Bascom et al., 2018) and the oscillations of the cytoplasmic Ca^2+^ concentration as a second messenger are involved in various physiological reactions and signal transmission processes (Sanders et al., 1999; Thor, 2019). So far, only a few extracellular mediators have been found to affect cytoplasmic Ca^2+^ “signatures”. In Arabidopsis, *AtRALF1* increased the cytoplasmic Ca^2+^ level by promoting the influx of extracellular Ca^2+^ and reducing the efflux of intracellular Ca^2+^ (Haruta et al., 2008). Later, the relationship between RALF-induced Ca^2+^ signal and pH was confirmed as RALF-induced extracellular pH change depends on Ca^2+^ signal, which occurs before alkalinization (Gjetting et al., 2020). *AtRALF1* also interacts in a Ca^2+^- and pH-dependent manner with calmodulin-like 38 for regulating root growth (Campos et al., 2018). Furthermore, RALF33 treatment did not affect the characteristics of Ca^2+^ and H^+^ while RALFL36 treatment showed some effects in the *fer* Arabidopsis mutant (Gjetting et al., 2020), again suggesting that different RALF peptides may bind with different receptors to trigger intracellular Ca^2+^, which later may activate H^+^ pumping at PM. Therefore, exploring other unknown RALF receptor-induced Ca^2+^ oscillations and testing whether their signal pathways overlap is a direction noteworthy in the future (Tanveer et al., 2018; Choudhary et al., 2021).

RALF proteins regulate the overall plant growth and redox homeostasis by regulating ROS production (Zhang X. et al., 2020). For instance, FER positively regulates root hair polar growth by regulating auxin-mediated ROS production (Yu et al., 2012). Likewise, FER and related proteins regulate ROS production by regulating the transcription of respiratory burst oxidase homologs (RBOH) (Franck et al., 2018). For instance, the FER–LLG1–Rop–Guanine Nucleotide Exchange factor complex regulates RBOH dependence ROS production (Duan et al., 2010; Li et al., 2015). ANX1 and ANX2 also maintain ROS production and regulate CW integrity during pollen tube growth (Boisson-Dernier et al., 2013); thus, RALFs protein complex requires ROS as important signaling molecules for regulating cell growth (Zhang X. et al., 2020). Nonetheless, the overproduction of ROS under SS is inevitable, and ST is linked to maintaining an equilibrium between overall ROS production and ROS scavenging; thus, tight regulation is required (Tanveer and Shabala, 2018; Khan et al., 2020). Moreover, the effects of SS on the overall redox state are highly tissue-specific and NaCl dose-specific (Shah et al.). This should be explored in future studies.

CW biosynthesis is a very complex mechanism in plants, and to examine the CW status, plants exhibit the CW integrity maintenance (CWIM) system. The CWIM system assists plants in adapting to stress conditions without compromising the integrity and organization of CW (Liu et al., 2021). SS can adversely affect CWI; thus, plants’ ability to maintain the CWIM system is essential for ST. Having said that, Ca^2+^ being a universal secondary messenger is actively involved in the operation of CWIM system in plants. However, the maintenance of balance between Ca^2+^ concentrations in CW, apoplast, and cytoplasm raises the question relating to the validity of this concept. In this context, Feng et al. (2018) showed that FER is required for the activation of Ca^2+^ influx and maintenance of CWI under salt stress (**Figure 1B**). FER is an important CWIM sensor and required for Ca2+ influx into cytoplasm under SS (Feng et al., 2018). FER contains two malectin domains that directly bind with de-methyl-esterified HG *in vitro* and *in vivo* (Feng et al., 2018; Lin et al., 2018; Liu et al., 2021). This suggests that FER probably senses the CW changes directly via its extracellular domain and then transduces the CW signals to the cytoplasm via its cytoplasmic kinase domain. Later, it was shown that SS may dissociate the LRX3/4/5-RALFs complex via the SS-induced ROS and pH changes in the apoplast, and the released RALFs bind to the LLG1–FER complex and thereby allow the transduction of CW signals (Zhao et al., 2018). The mechanism behind the dissociation of LRX3/4/5 and RALFs under SS needs to be further investigated. Moreover, FEI1 and FEI2 are two other important LRR–RLK complexes that regulate cellulose synthesis in the CW (Xu et al., 2008), and loss-of-function mutants of FEI1 and FEI2 showed roots with reduced cellulose contents (Basu et al., 2015), thus indicating the role of FEIs in CWI sensing.

Hormonal regulation under SS is also important, as different phytohormones regulate different physiological processes (Tanveer et al., 2018; Choudhary et al., 2021). Abscisic acid (ABA) is an essential hormone of plant stress resistance and tolerance. The signal crosstalk between RALF and ABA is involved in the response of plants to abiotic stresses including SS and water deficit (Chen et al., 2016). Studies showed that the RALF1-FER signaling pathway activates ABI2 (ABA Insensitive 2) phosphatase by the GEF1/4/10-ROP11 pathway and further inhibits ABA response (Yu et al., 2012; Chen et al., 2016). The LRX is an important receptor of CWI signal, and the *LRX3*/*4*/*5* triple mutants as well as FER mutants displayed salt hypersensitivity, which was mimicked by overexpression of RALF22/23 (Zhao et al., 2018). In Arabidopsis, LRX3/4/5-RALF22/23-FER regulated ST by regulating equilibrium between ROS production and accumulation of phytohormones (ABA, JA, and SA) (Zhao et al., 2021). Moreover, RALF acts as upstream of the ROS regulatory pathway and has been shown to interact with ABA to regulate the growth of plant roots (Chen et al., 2016).

The AGB1-a G protein β-subunit involved in ABA mediating stomatal opening and FER-ABG1 are reported to be involved in salt stress responses (Yu et al., 2018a). Mutants lacking FER and ABG1 showed a hypersensitive phenotype to salt stress (Yu et al., 2018a), and even the application of *AtRALF* was not effective in reducing hypersensitivity to salt stress (Zhao et al., 2018). In contrast, *AtRALF1* mutants did not show any response to higher salt stress levels (Feng et al., 2018). Taking an example of non-vascular plants, *PpRALF3* knockout lines showed higher resistance under SS and ROS stress in moss (*Physcomitrium patens*), implying the functional role of RALFs in regulating ST (Solís-Miranda et al., 2023). However, the relationship of RALFs with other phytohormones, e.g., melatonin, should be considered in future studies. Melatonin-induced enhancement of PM H^+^-ATPase activity may negate salinity-induced MP depolarization, preventing the activation of outward K^+^ channels and thereby leading to higher ST (Yu et al., 2018b). Moreover, the abiotic stress regulatory role of MEL has also been reported elsewhere (Tanveer and Shabala, 2020; Huang et al., 2022, 2024).

Given that, RALFs’ gene expression is highly specific to plant species and tissues (Kim et al., 2021). For instance, five homologs of RALFs were observed in poplar (Haruta and Constabel, 2003) while 37 homologs were in Arabidopsis (Abarca et al., 2021). A total of 765 RALF proteins were identified from 51 plant species (Campbell and Turner, 2017). Recently, a genome-wide association study revealed 163 RALF genes in seven species from the Rosaceae family, including 45 mature RALF genes (Zhang H. et al., 2020). A phylogenetic tree analysis showed the diversification of different RALF genes in different plant species (**Figure 1C**), thus indicating that the genetic differences among different plant species could also govern the regulation of RALF genes in plants.

## Conclusions

RALFs are widely distributed in plants and RALF-induced cell expansion is the result of the interaction of the changes of intra- and extracellular ions, deposition of new CW materials, and the rearrangement of existing CWs. During the transition from vegetative growth to reproductive growth, RALFs have different functions in maintaining the normal life activities of plants. This is achievable because RALF forms a complex signal network to complete these complex functions. For instance, the RALF–FER signal transduction pathway is highly conserved in plants and is very important for mediating RALF signaling. Thus, the physiological significance of the regulatory activities of RALF and some membrane receptors is still unknown and several outstanding questions should be focused on while examining the role of RALFs in regulating ST in plants. For instance,

- How RALF-induced FER-specific internalization conducts Ca^2+^ transduction in CW is also a focus of research.
- Equally interesting is the question of intracellular signal transduction of RALF-induced FER-specific internalization. While it is widely accepted that RALFs regulate plant growth and development together with CW components and PM receptors, spatiotemporal aspects of such regulation remain largely unknown, in the light of the apparent dual function of RALF.
- It is also worth noting that some plant hormones such as ABA or melatonin are also involved in ST; however, whether RALF is directly or indirectly involved in crosstalk with these phytohormones to mediate ST is not fully comprehended.
- Moreover, whether RALF is directly or indirectly involved in ion rebalance and transport under SS via regulating Ca^2+^ signaling and H^+^-ATPase is not yet clear. Therefore, exploring the mechanism of RALF under SS is also an important direction and a great challenge for future research in developing ST crops.
- Given that the RALF peptide family is very diverse and binds to large arrays of receptors, mechanisms regulating the specificity of the RALF–receptor interaction under different growth conditions should be examined.

## References

[B1] Abarca A. Franck C. M. Zipfel C. (2021). Family-wide evaluation of rapid alkalinization factor peptides. Plant Physiol. 187, 996–1010. doi: 10.1093/plphys/kiab308 34608971 PMC8491022

[B2] Bascom C. S. Hepler P. K. Bezanilla M. (2018). Interplay between ions, the cytoskeleton, and cell wall properties during tip growth. Plant Physiol. 176, 28–40. doi: 10.1104/pp.17.01466 29138353 PMC5761822

[B3] Basu D. Wang W. Ma S. DeBrosse T. Poirier E. Emch K.. (2015). Two hydroxyproline galactosyltransferases, GALT5 and GALT2, function in arabinogalactan-protein glycosylation, growth and development in Arabidopsis. PloS One 10(5), e0125624. doi: 10.1371/journal.pone.0125624 25974423 PMC4431829

[B4] Blackburn M. R. Haruta M. Moura D. S. (2020). Twenty years of progress in physiological and biochemical investigation of RALF peptides. Plant Physiol. 182, 1657–1666. doi: 10.1104/pp.19.01310 32071151 PMC7140910

[B5] Boisson-Dernier A. Lituiev D. S. Nestorova A. Franck C. M. Thirugnanarajah S. Grossniklaus U. (2013). ANXUR receptor-like kinases coordinate cell wall integrity with growth at the pollen tube tip via NADPH oxidases. PloS Biol. 11, e1001719. doi: 10.1371/journal.pbio.1001719 24302886 PMC3841104

[B6] Bose J. Rodrigo-Moreno A. Lai D. Xie Y. Shen W. Shabala S. (2015). Rapid regulation of the plasma membrane H^+^-ATPase activity is essential to salinity tolerance in two halophyte species, Atriplex lentiformis and Chenopodium quinoa. Ann. Bot. 115, 481–494. doi: 10.1093/aob/mcu219 25471095 PMC4332608

[B7] Campbell L. Turner S. R. (2017). A comprehensive analysis of RALF proteins in green plants suggests there are two distinct functional groups. Front. Plant Sci. 8. doi: 10.3389/fpls.2017.00037 PMC5258720 28174582

[B8] Campos W. F. Dressano K. Ceciliato P. H. O. Guerrero-Abad J. C. Silva A. L. Fiori C. S.. (2018). *Arabidopsis thaliana* rapid alkalinization factor 1–mediated root growth inhibition is dependent on calmodulin-like protein 38. J. Biol. Chem. 293, 2159–2171. doi: 10.1074/jbc.m117.808881 29282286 PMC5808775

[B9] Cao J. Shi F. (2012). Evolution of the RALF gene family in plants: gene duplication and selection patterns. Evol. Bioinf. 8, EBO.S9652. doi: 10.4137/ebo.s9652 PMC3382376 22745530

[B10] Chakraborty K. Bose J. Shabala L. Shabala S. (2016). Difference in root K^+^ retention ability and reduced sensitivity of K+ -permeable channels to reactive oxygen species confer differential salt tolerance in three Brassica species. J. Exp. Bot. 67, 4611–4625. doi: 10.1093/jxb/erw236 27340231 PMC4973732

[B11] Chen J. Yu F. Liu Y. Du C. Q. Li X. S. Zhu S. R.. (2016). FERONIA interacts with ABI2-type phosphatases to facilitate signaling cross-talk between abscisic acid and RALF peptide in *Arabidopsis* . Proc. Natl. Acad. Sci. 113, E5519–E5527. doi: 10.1073/pnas.1608449113 PMC5027425 27566404

[B12] Chen F. Yuan Y. Li Q. He Z. (2007). Proteomic analysis of rice plasma membrane reveals proteins involved in early defense response to bacterial blight. PROTEOMICS 7, 1529–1539. doi: 10.1002/pmic.200500765 17407182

[B13] Choudhary P. Pramitha L. Rana S. Verma S. Aggarwal P. R. Muthamilarasan M. (2021). Hormonal crosstalk in regulating salinity stress tolerance in graminaceous crops. Physiol. Plant. 173, 1587–1596. doi: 10.1111/ppl.13558 34537966

[B14] Duan Q. Kita D. Li C. Cheung A. Y. Wu H. M. (2010). FERONIA receptor-like kinase regulates RHO GTPase signaling of root hair development. Proc. Natl. Acad. Sci. U.S.A. 107, 17821–17826. doi: 10.1073/pnas.1005366107 20876100 PMC2955125

[B15] Feng W. Kita D. Peaucelle A. Cartwright H. N. Doan V. Duan Q.. (2018). The FERONIA receptor kinase maintains cell-wall integrity during salt stress through Ca^2+^ signaling. Curr. Biol. 28, 666–675. doi: 10.1016/j.cub.2018.01.023 29456142 PMC5894116

[B16] Franck C. M. Westermann J. Boisson-Dernier A. (2018). Plant malectin-like receptor kinases: from cell wall integrity to immunity and beyond. Annu. Rev. Plant Biol. 69 (2018), 301–328. doi: 10.1146/annurev-arplant-042817-040557 29539271

[B17] Ge Z. Bergonci T. Zhao Y. Zou Y. J. Du S. Liu M. C.. (2017). *Arabidopsis* pollen tube integrity and sperm release are regulated by RALF-mediated signaling. Science 358, 1596–1600. doi: 10.1126/science.aao3642 29242234 PMC5964610

[B18] Ge Z. Cheung A. Y. Qu L. (2019). Pollen tube integrity regulation in flowering plants: insights from molecular assemblies on the pollen tube surface. New Phytol. 222, 687–693. doi: 10.1111/nph.15645 30556141

[B19] Ginanjar E. F. Teh O. Fujita T. (2022). Characterisation of rapid alkalinisation factors in *Physcomitrium patens* reveals functional conservation in tip growth. New Phytol. 233, 2442–2457. doi: 10.1111/nph.17942 34954833

[B20] Gjetting S. K. Mahmood K. Shabala L. Kristensen A. Shabala S. Fuglsang A. T. (2020). Evidence for multiple receptors mediating RALF-triggered Ca^2+^ signaling and proton pump inhibition. Plant J. 104, 433–446. doi: 10.1111/tpj.14935 32713048

[B21] Haruta M. Constabel C. P. (2003). Rapid alkalinization factors in poplar cell cultures. Peptide Isolation, cDNA Cloning, and Differential Expression in Leaves and Methyl Jasmonate-Treated Cells. Plant Physiol. 131, 814–823. doi: 10.1104/pp.014597 12586905 PMC166857

[B22] Haruta M. Monshausen G. Gilroy S. Sussman M. R. (2008). A cytoplasmic Ca^2+^ functional assay for identifying and purifying endogenous cell signaling peptides in *Arabidopsis* Seedlings: identification of AtRALF1 peptide. Biochemistry 47, 6311–6321. doi: 10.1021/bi8001488 18494498

[B23] Haruta M. Sabat G. Stecker K. Minkoff B. B. Sussman M. R. (2014). A peptide hormone and its receptor protein kinase regulate plant cell expansion. Science 343, 408–411. doi: 10.1126/science.1244454 24458638 PMC4672726

[B24] Huang L. Fu W. Zhang Y. Liu X. Wang Q. Wang L.. (2024). The role of melatonin in regulating horticultural crop production under various abiotic stresses. Scientia Horticult. 323, 112508. doi: 10.1016/j.scienta.2023.112508

[B25] Huang X. Tanveer M. Min Y. Shabala S. (2022). Melatonin as a regulator of plant ionic homeostasis: implications for abiotic stress tolerance. J. Exp. Bot. 73, 5886–5902. doi: 10.1093/jxb/erac224 35640481

[B26] Kesten C. Wallmann A. Schneider R. McFarlane H. E. Diehl A. Khan G. A.. (2019). The companion of cellulose synthase 1 confers salt tolerance through a Tau-like mechanism in plants. Nat. Commun. 10, 857. doi: 10.1038/s41467-019-08780-3 30787279 PMC6382854

[B27] Khan W. U. D. Tanveer M. Shaukat R. Ali M. Pirdad F. (2020). An overview of salinity tolerance mechanism in plants. Salt drought Stress tolerance plants: Signaling Networks adaptive mechanisms, 1–16. doi: 10.1007/978-3-030-40277-8_1

[B28] Kim D. Yang J. Gu F. Park S. Combs J. Adams A.. (2021). A temperature-sensitive FERONIA mutant allele that alters root hair growth. Plant Physiol. 185, 405–423. doi: 10.1093/plphys/kiaa051 33721904 PMC8133571

[B29] Li L. Chen H. Alotaibi S. S. Pěnčík A. Adamowski M. Novák O.. (2022a). RALF1 peptide triggers biphasic root growth inhibition upstream of auxin biosynthesis. Proc. Natl. Acad. Sci. 119, e2121058119. doi: 10.1073/pnas.2121058119 35878023 PMC9351349

[B30] Li C. Yeh F. L. Cheung A. Y. Duan Q. Kita D. Liu M. C.. (2015). Glycosylphosphatidylinositol-anchored proteins as chaperones and co-receptors for FERONIA receptor kinase signaling in Arabidopsis. eLife 4, e06587. doi: 10.7554/elife.06587 26052747 PMC4458842

[B31] Li Y. Zeng H. Xu F. Yan F. Xu W. (2022b). H^+^-ATPases in plant growth and stress responses. Annu. Rev. Plant Biol. 73, 495–521. doi: 10.1146/annurev-arplant-102820-114551 35231180

[B32] Lin W. Tang W. Anderson C. T. Yang Z. (2018). FERONIA’s sensing of cell wall pectin activates ROP GTPase signaling in Arabidopsis. BioRxiv, 269647. doi: 10.1101/269647

[B33] Liu L. Liu X. Bai Z. Tanveer M. Zhang Y. Chen W.. (2024). Small but powerful: RALF peptides in plant adaptive and developmental responses. Plant Sci. 343, 112085. doi: 10.1016/j.plantsci.2024.112085 38588983

[B34] Liu J. Zhang W. Long S. Zhao C. (2021). Maintenance of cell wall integrity under high salinity. Int. J. Mol. Sci. 22, 3260. doi: 10.3390/ijms22063260 33806816 PMC8004791

[B35] Murphy E. Vu L. D. Broeck L. Zhefeng Lin Z. F. Ramakrishna P. Cotte B.. (2016). RALFL34 regulates formative cell divisions in Arabidopsis pericycle during lateral root initiation. J. Exp. Bot. 67, 4863–4875. doi: 10.1093/jxb/erw281 27521602 PMC4983113

[B36] Palmgren M. G. Nissen P. (2011). P-type ATPases. Annu. Rev. Biophys. 40, 243–266. doi: 10.1146/annurev.biophys.093008.131331 21351879

[B37] Pearce G. Moura D. S. Stratmann J. Ryan C. A. (2001). RALF, a 5-kDa ubiquitous polypeptide in plants, arrests root growth and development. Proc. Natl. Acad. Sci. 98, 12843–12847. doi: 10.1073/pnas.201416998 11675511 PMC60141

[B38] Rubio F. Nieves-Cordones M. Horie T. Shabala S. (2020). Doing ‘business as usual’comes with a cost: evaluating energy cost of maintaining plant intracellular K+ homeostasis under saline conditions. New Phytol. 225 ((3)), 1097–1104. doi: 10.1111/nph.15852 30993727

[B39] Sanders D. Brownlee C. Harper J. F. (1999). Communicating with calcium. Plant Cell. 11, 691. doi: 10.1105/tpc.11.4.691 10213787 PMC144209

[B40] Shabala S. Bose J. Fuglsang A. T. Pottosin I. (2016). On a quest for stress tolerance genes: membrane transporters in sensing and adapting to hostile soils. J. Exp. Bot. 67, 1015–1031. doi: 10.1093/jxb/erv465 26507891

[B41] Shabala S. Wu H. Bose J. (2015). Salt stress sensing and early signalling events in plant roots: Current knowledge and hypothesis. Plant Sci. 241, 109–119. doi: 10.1016/j.plantsci.2015.10.003 26706063

[B42] Shabala L. Zhang J. Pottosin I. Bose J. Zhu M. Fuglsang A. T.. (2016). Cell-type-specific H+-ATPase activity in root tissues enables K+ retention and mediates acclimation of barley (Hordeum vulgare) to salinity stress. Plant Physiol. 172 (4), 2445–2458. doi: 10.1104/pp.16.01347 27770060 PMC5129721

[B43] Sharma A. Hussain A. Mun B.-G. Imran Q. M. Falak N. Lee S. U.. (2016). Comprehensive analysis of plant rapid alkalization factor (RALF) genes. Plant Physiol. Biochem. 106, 82–90. doi: 10.1016/j.plaphy.2016.03.037 27155375

[B44] Solís-Miranda J. Juárez-Verdayes M. A. Nava N. Rosas P. Leija-Salas A. Cárdenas L.. (2023). The phaseolus vulgaris receptor-like kinase pvFER1 and the small peptides pvRALF1 and pvRALF6 regulate nodule number as a function of nitrate availability. Int. J. Mol. Sci. 24, 5230. doi: 10.3390/ijms24065230 36982308 PMC10049175

[B45] Tanveer M. Ahmed H. A. I. (2020). ROS signalling in modulating salinity stress tolerance in plants. Salt Drought Stress Tolerance Plants: Signaling Networks Adapt. Mechan., 299–314. doi: 10.1007/978-3-030-40277-8_11

[B46] Tanveer M. Shabala S. (2018). Targeting Redox Regulatory Mechanisms for Salinity Stress Tolerance in Crops. Salinity Responses and Tolerance in Plants 1, 213–234. doi: 10.1007/978-3-319-75671-4_8

[B47] Tanveer M. Shabala S. (2020). Neurotransmitters in signalling and adaptation to salinity stress in plants. Neurotransmit. Plant Signaling commun., 49–73. doi: 10.1007/978-3-030-54478-2_3

[B48] Tanveer M. Shahzad B. Sharma A. Biju S. Bhardwaj R. (2018). 24-Epibrassinolide; an active brassinolide and its role in salt stress tolerance in plants: A review. Plant Physiol. Biochem. 130, 69–79. doi: 10.1016/j.plaphy.2018.06.035 29966934

[B49] Thor K. (2019). Calcium—Nutrient and messenger. Front. Plant Sci. 10. doi: 10.3389/fpls.2019.00440 PMC6495005 31073302

[B50] Wegner L. H. Stefano G. Shabala L. Rossi M. Mancuso S. Shabala S. (2011). Sequential depolarization of root cortical and stelar cells induced by an acute salt shock–implications for Na^+^ and K^+^ transport into xylem vessels. Plant Cell Environ. 34 (5), 859–869. doi: 10.1111/j.1365-3040.2011.02291.x 21332511

[B51] Wu J. Kurten E. L. Monshausen G. Hummel G. M. Gilroy S. Baldwin I. T. (2007). NaRALF, a peptide signal essential for the regulation of root hair tip apoplastic pH in *Nicotiana attenuata*, is required for root hair development and plant growth in native soils. Plant J. 52, 877–890. doi: 10.1111/j.1365-313x.2007.03289.x 17916115

[B52] Xiao Y. Stegmann M. Han Z. DeFalco T. A. Parys K. Xu L.. (2019). Mechanisms of RALF peptide perception by a heterotypic receptor complex. Nature 572, 270–274. doi: 10.1038/s41586-019-1409-7 31291642

[B53] Xu S. L. Rahman A. Baskin T. I. Kieber J. J. (2008). Two leucine-rich repeat receptor kinases mediate signaling, linking cell wall biosynthesis and ACC synthase in Arabidopsis. Plant Cell 20 (11), 3065–3079. doi: 10.1105/tpc.108.063354 19017745 PMC2613664

[B54] Yu Y. Chakravorty D. Assmann S. M. (2018a). The G protein *β*-subunit, AGB1, interacts with FERONIA in RALF1-regulated stomatal movement. Plant Physiol. 176, 2426–2440. doi: 10.1104/pp.17.01277 29301953 PMC5841690

[B55] Yu F. Qian L. Nibau C. Duan Q. B. Kita D. Levasseur K.. (2012). FERONIA receptor kinase pathway suppresses abscisic acid signaling in *Arabidopsis* by activating ABI2 phosphatase. Proc. Natl. Acad. Sci. 109, 14693–14698. doi: 10.1073/pnas.1212547109 22908257 PMC3437822

[B56] Yu Y. Wang A. Li X. Kou M. Wang W. Chen X.. (2018b). Melatonin-stimulated triacylglycerol breakdown and energy turnover under salinity stress contributes to the maintenance of plasma membrane H^+^–ATPase activity and K^+^/Na^+^ homeostasis in sweet potato. Front. Plant sci. 9. doi: 10.3389/fpls.2018.00256 PMC5835075 29535758

[B57] Zhang H. Jing X. Chen Y. Liu Z. Xin Y. Qiao Y. (2020). The genome-wide analysis of RALF-like genes in strawberry (wild and cultivated) and five other plant species (Rosaceae). Genes 11, 174. doi: 10.3390/genes11020174 32041308 PMC7073784

[B58] Zhang X. Yang Z. Wu D. Yu F. (2020). RALF–FERONIA signaling: linking plant immune response with cell growth. Plant Commun. 1 (4), 100084. doi: 10.1016/j.xplc.2020.100084 33367248 PMC7747976

[B59] Zhao C. Jiang W. Zayed O. Xin Liu X. Tang K. Nie W. F.. (2021). The LRXs-RALFs-FER module controls plant growth and salt stress responses by modulating multiple plant hormones. Natl. Sci. Review. 8, nwaa149. doi: 10.1093/nsr/nwaa149 PMC8288382 34691553

[B60] Zhao C. Zayed O. Yu Z. Jiang W. Zhu P. Hsu C. C.. (2018). Leucine-rich repeat extensin proteins regulate plant salt tolerance in *Arabidopsis* . Proc. Natl. Acad. Sci. 115, 13123–13128. doi: 10.1073/pnas.1816991115 30514814 PMC6305001

